# Smart ECM-Based Electrospun Biomaterials for Skeletal Muscle Regeneration

**DOI:** 10.3390/nano10091781

**Published:** 2020-09-09

**Authors:** Sara Politi, Felicia Carotenuto, Antonio Rinaldi, Paolo Di Nardo, Vittorio Manzari, Maria Cristina Albertini, Rodolfo Araneo, Seeram Ramakrishna, Laura Teodori

**Affiliations:** 1 Department of Fusion and Technologies for Nuclear Safety and Security, Diagnostic and Metrology (FSN-TECFIS-DIM), ENEA, CR Frascati, 00044 Rome, Italy; sara.politi@uniroma2.it (S.P.); carotenuto@med.uniroma2.it (F.C.); 2 Department of Clinical Science and Translational Medicine, University of Rome “Tor Vergata”, 00133 Rome Italy; dinardo@uniroma2.it (P.D.N.); manzari@uniroma2.it (V.M.); 3 Interdepartmental Center for Regenerative Medicine (CIMER), University of Rome “Tor Vergata”, 00133 Rome, Italy; 4 Department of Sustainability (SSPT), ENEA, 00123 Rome, Italy; antonio.rinaldi@gmail.com; 5 L.L. Levshin Institute of Cluster Oncology, I. M. Sechenov First Medical University, Moscow 119991, Russia; 6 Department of Biomolecular Sciences, Urbino University “Carlo Bo”, 61029 Urbino, Italy; maria.albertini@uniurb.it; 7 Department of Astronautics Electrical and Energy Engineering (DIAEE), University of Rome “La Sapienza”, 00184 Rome, Italy; rodolfo.araneo@uniroma1.it; 8 Centre for Nanofibers and Nanotechnology, Department of Mechanical Engineering, National University of Singapore, Singapore 119260, Singapore; seeram@nus.edu.sg

**Keywords:** smart biomaterials, electrospinning, biofunctionalization, decellularized extracellular matrix (dECM), skeletal muscle regeneration, click chemistry

## Abstract

The development of smart and intelligent regenerative biomaterials for skeletal muscle tissue engineering is an ongoing challenge, owing to the requirement of achieving biomimetic systems able to communicate biological signals and thus promote optimal tissue regeneration. Electrospinning is a well-known technique to produce fibers that mimic the three dimensional microstructural arrangements, down to nanoscale and the properties of the extracellular matrix fibers. Natural and synthetic polymers are used in the electrospinning process; moreover, a blend of them provides composite materials that have demonstrated the potential advantage of supporting cell function and adhesion. Recently, the decellularized extracellular matrix (dECM), which is the noncellular component of tissue that retains relevant biological cues for cells, has been evaluated as a starting biomaterial to realize composite electrospun constructs. The properties of the electrospun systems can be further improved with innovative procedures of functionalization with biomolecules. Among the various approaches, great attention is devoted to the “click” concept in constructing a bioactive system, due to the modularity, orthogonality, and simplicity features of the “click” reactions. In this paper, we first provide an overview of current approaches that can be used to obtain biofunctional composite electrospun biomaterials. Finally, we propose a design of composite electrospun biomaterials suitable for skeletal muscle tissue regeneration.

## 1. Introduction

Biomaterials play a prominent role in regenerative medicine and tissue engineering (TE) through the development of functional systems to improve or restore biological functions of damaged tissues. The current research focuses on the design of stimuli-responsive, smart, and intelligent biomaterials systems that are able to modulate their physical, chemical, and mechanical properties in response to external chemical or physical stimuli and changes in the physiological environments adapting their functionality accordingly.

The development of regenerative biomaterials and the progress in their processing represent key factors to generate smart biomimetic scaffolds resembling the structural organization and activity of native tissue in order to guide tissue regeneration [1,2].

Nowadays, researchers aim to develop tissue-specific scaffolds characterized by desired topographical mechanical and physical features. Among them, the skeletal muscle represents a complex and challenging tissue to be generated in vitro for tissue engineering purposes.

Skeletal muscle is a complex system of oriented muscle fibers acting together to produce a contractile force and support body movement. In addition, the proper function of skeletal muscle encompasses breathing, metabolic control, thermoregulation, and energy storage [3]. Skeletal muscle has innate regenerative potential following injury or disease. However, endogenous self-repair is severely impaired due to volume traumatic muscle loss.

Severe traumatic injuries of skeletal muscles are responsible for functional deficits in patients. Owing to the wide prevalence of these injuries and the associated socio-economic implications, muscle regeneration has been a topic of scientific and clinical interests [4]. The potential strategies for designing a suitable biomaterial for skeletal muscle regeneration should take into account important physical, biochemical, and inflammatory cues that effectively affect cell adhesion and proliferation and thus guide muscle regeneration. Among the most important properties that biomaterials should possess for successful skeletal muscle TE include: porosity, aligned architecture, and bioavailability of bioactive molecules to promote the cell activity [3].

Many studies have demonstrated that the advanced manufacturing of biomimetic scaffolds is a crucial aspect of a successful regenerative process [5,6]. Scaffolding strategies aim to create ideal scaffolds with mechanical, chemical, and biological properties that mimic the composition and the structure of extracellular matrix (ECM) of native tissue to encourage cell adhesion and proliferation [7,8]. The ECM represents the major structural component of the human body, and it is composed of a three-dimensional arrangement of natural polymers such as collagen, elastin, and fibrinogen as well as a mixture of macromolecules such as growth factors, peptides, glycoproteins, and proteoglycans. However, the ECM is not only a structural framework for tissues but through interactions with receptors on the surfaces of cells, it plays an important role in both day-to-day cellular activity and in tissue regeneration [9,10]. For these reasons, the engineering of an ECM-mimicking scaffold is extremely challenging.

In the native tissues, most ECM components consist of interwoven fibrous structures in the micronanoscale range and thus, the fabrication of scaffolds mimicking ECM structural organization is an active area of research in TE. To date, phase separation, self-assembly, and electrospinning have been used to make scaffolds with a fibrous network [11]. Among these techniques, electrospinning has continued to be the most commonly used.

Electrospinning is a powerful and scalable production method [12], which allows the fabrication of micro- or nanofibers possessing large surface areas and high porosity, which are favorable for biomedical applications in terms of cellular interactions [13,14].

A typical electrospinning set-up consists of a high-voltage power supply, a syringe with a needle, and an electrically conductive collector. During the process, a high voltage is applied to the needle of a syringe, which contains a polymeric solution, and a collector. Once the electric field reaches a critical value at which the repulsive electric force overcomes the surface tension of polymeric solution, this is ejected from the syringe needle. The fibers are formed during the fast evaporation of the solvent and are deposited on the collector [14].

The advantages of electrospinning for scaffold fabrication are its versatility, flexibility, the possibility to use different materials combinations, and malleability to conform over a wide variety of form factors (e.g., sizes and shapes) [15].

However, during a typical electrospinning process, fibers are densely deposited on a collector forming closed packed fibers that are associated with poor cell infiltration. To date, many interesting approaches are investigated to enhance porosity in electrospun scaffolds and to acquire pores of the suitable size in order to obtain greater cell infiltration [3,16].

In particular, to control the fiber arrangement, topography, morphology, and the overall performance of electrospun polymeric scaffolds for TE applications, different electrospinning processing factors, such as applied voltage, tip of needle-collector distance, solution viscosity, and feed rate need to be optimized [17]. The role of main effects and interactions between process parameters can be effectively captured by regression and design of experiments methods [18,19]. Randomly oriented or aligned fibers can be formed by using a stationary or rotating collector, respectively. The orientation of fibers is an important feature of an ideal and promising scaffold because this aspect greatly influences cell growth and related functions in cells such as nerve and smooth muscle cells [20]. In particular, the creation of polymeric aligned electrospun biomaterials that mimic skeletal muscle allows the efficient organization of muscle cells to form aligned myotubes during muscle regeneration [3]. 

Over the past two decades, electrospun scaffolds have been obtained using natural polymers, synthetic polymers, or a combination of those [21,22,23] according to the desired combination of mechanical and chemical properties that ultimately dictate the biological response toward the targeted tissue regeneration application.

Recently, the development of the decellularization processes of organs and tissues has made possible the creation of an electrospun scaffold prepared from processing methods of a native extracellular matrix. The decellularized extracellular matrix (dECM) is an organ or a tissue devoid of its cellular content but which preserves a rather large portion of its original composition including its architecture, structural organization, and biochemical cues [24,25].

This dECM material can be used as a scaffold that maintains its original geometry directly in medical interventions or can be repopulated with cells before use. More recently, dECMs have been further processed to generate dECM products that can be used as a starting material for biofabrication techniques such as electrospinning [26]. Indeed, electrospun dECM scaffolds are typically achieved by adding components of dECM after the electrospinning process or by direct electrospinning dECM components [27]. However, during processing to obtain the dECM materials, some key components such as glycosaminoglycans (GAGs) can be lost. Indeed, GAG concentrations are especially important for their interaction with growth factors and chemokines, influencing cell signaling [28].

To overcome these obstacles, some authors are developing ad hoc decellurization protocols for specific tissues and the optimization of subsequent dECM process methods with the aim of avoiding the loss of some important bioactive components of the native ECM [29,30].

Another effective way to ensure the bioactivity of the electrospun scaffolds is the judicious combination of electrospun fibers with appropriate biomolecules, such as small molecules, growth factors, short peptides, or proteins [31], in order to stimulate a specific cell response.

In this context, the development of innovative and forefront electrospinning techniques and an accurate choice of polymeric materials and biomolecules enables the loading of bioactive components during the manufacturing process. A local and controlled release of biomolecules enhances the functional properties of scaffold and influence surrounding tissue regeneration.

Furthermore, the regeneration process is strongly influenced by the interactions between cells and biomaterial surfaces. For this reason, the postelectrospinning modification of the high surface of fibers with biomolecules represents a suitable method in order to enhance cell adhesion and organization [22,32]. Such postelectrospinning modifications can be achieved by the physical adsorption or chemical bonding of biomolecules to the fibers. In this context, the biofunctionalization of the fiber surface using “click” reactions has drawn significant interest recently, owing to their simple reaction conditions, high reaction rate, and high chemical selectivity [33,34].

**Figure 1:**
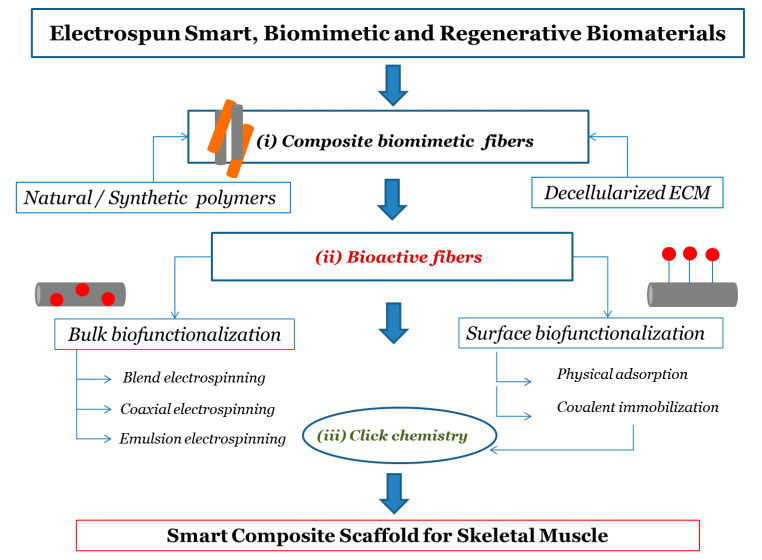
Flow chart for designing process of smart electrospun scaffold for skeletal muscle regeneration. The main steps concerns (i) composite electrospun biomaterials; (ii) the incorporation (bulk biofunctionalization) and surface attachment of bioactive molecules (surface biofunctionalization) during and after electrospinning process, respectively; (iii) surface biofunctionalization postprocessing using “click” reactions.

In this paper, we discuss current approaches that can be used to obtain functional composite electrospun biomaterials with a focus on the possible strategies to achieve bioactive scaffolds. The main steps discussed concern (i) obtaining composite electrospun biomaterials with particular attention to the use of native ECM in the solution or fusion of electrospinning; (ii) the incorporation of bioactive molecules during electrospinning (bulk functionalization) and the surface modification of electrospun systems through biomolecules; (iii) biofunctionalization postprocessing using “click” reactions, as schematically represented in Figure 1.

Finally, we designed a strategy for dECM-based composite bioactive electrospun scaffolds to promote skeletal muscle regeneration.

## 2. Biomaterials for Electrospinning in Tissue Engineering

### 2.1. Synthetic and Natural Polymers

The choice of polymeric materials is the first design step for the electrospun scaffold. Natural and synthetic polymers have been widely used to realize scaffolds due to their properties such as biodegradation, mechanical properties, high porosity and surface to volume ratio, and also small pore size [8,35].

In particular, the growing success of synthetic polymers as scaffolding materials is due to their facile synthesis and processing flexibility, enabling good reproducibility and tuneable characteristics. Polyesters (e.g., poly(ε-caprolactone) (PCL), polylactic acid (PLA), polyglycolic acid (PGA), etc.), and polyethers (e.g., polyethylene oxide (PEO), polyurethane (PU), etc.) have been electrospun for TE applications [21,22]. Furthermore, electrospun fibers made of shape memory polymers (SMPs) have also been obtained thanks to the optimization of the experimental parameters [36].

Despite the excellent biodegradability, chemical, and mechanical properties of electrospun nanofibrous scaffolds from synthetic polymers, they often require further modification to their surface and structure to promote their biofunctionality, since synthetic biomaterials lack bioactive functional sites and result in poor biochemical similarities with ECM [37].

Conversely, compared to synthetic polymers, natural polymers offer intrinsic similarity to ECM components and bring with them a biological signature that is advantageous to support cell adhesion and proliferation [8,21,38]. Collagen, gelatin, chitosan, hyaluronic acid, cellulose, and glycans in general have in fact been proposed to produce electrospun fibers in TE because of their biocompatibility and low immunogenicity [21,39]. However, these scaffolds usually lack load-bearing capability due to lower mechanical strength and higher degradation rates than synthetic polymers [21,22].

It is worth noting from a manufacturing standpoint that the electrospinning of natural polymers is very often possible with water-based recipes and with minimal or no recourse to organic solvent, which is a definitive advantage in the ongoing societal transition to green chemistry and circular economy [40] and is only possible with a handful of synthetic counterparts (e.g., PEO, PVOH).

### 2.2. Composite Polymeric Electrospun Fibers

A blend of polymers can be an attractive option to mix the benefits and overcome the limitations of the individual one-component systems described above. In particular, the combination of natural and synthetic polymers is a means to effectively improve the biocompatibility, mechanical, and structural properties of the scaffold to boost cell adhesion and growth and to promote tissue regeneration [41].

A homogeneous blend composed of a selected mixture of natural and synthetic polymers can be designed and electrospun to fabricate composite scaffolds that are endowed with physicochemical properties of its components, such as hydrophilicity/hydrophobicity, surface charge, and mechanical strength of synthetic polymers as well as the biological features of natural fibers [22,23].

Focusing on binary compound systems, several attempts are found in literature, especially featuring the combination of PCL with another natural component. For example, Zhang et al. [42] fabricated a composite electrospun scaffold based on PCL and gelatin, which exhibited enhanced mechanical properties and wettability (vs. water) compared to those obtained from either PCL or gelatin alone. In addition, cell culture experiments highlighted favorable interactions between the composite scaffold and bone marrow stromal cells (BMSCs), suggesting the potential use of composite gelatin/PCL nanofibers in TE applications.

Other combinations of PCL with collagen [43] and chitosan [44] are reported. The results invariably showed an improvement in the biocompatibility and mechanical properties of the composite systems.

In general, electrospun PCL-based composites are characterized by modulable mechanical properties, namely: flexural strain, tensile strain, stiffness (i.e., Young’s modulus and in-plane stiffness at the mesoscale), and thermomechanical strength [18,45,46] for biomedical treatments, particularly important in the implantation of artificial bone and muscle tissues regeneration. In another study, PCL and collagen type I-based systems were investigated for creating implantable engineered muscle tissue that closely mimics the native tissue [47]. The results suggested that aligned PCL/collagen nanofibers facilitate skeletal muscle cell organization and myotube formation as compared to randomly oriented nanofibers. Moreover, the stiffness of the electrospun PCL-based composites makes these scaffolds promising for in-vivo applications [48].

Apart from PCL, other binary synthetic-natural systems are possible. Biomimetic and biocompatible nanofibrous scaffolds of polyamide-6,6 (PA 6,6) blended with chitosan were fabricated for applications in bone TE [49]. In particular, the effects of three different weight percentages of chitosan were investigated. The results showed that the increase in the concentration of chitosan enhances the performances of the composite scaffold in terms of cell growth, adhesion, differentiation, and proliferation.

Additionally, composite electrospun fibers containing gelatin and synthetic polymers exhibited attractive physicochemical, biomechanical, and biocompatibility properties that mimic the important features of natural ECM [50].

Other synthetic polymers such as poly(vinyl alcohol) (PVA) [51] and poly(lactic-co-glycolic acid) (PLGA) [52] were used in combination with natural polymers as electrospun scaffolds, reporting promising results in TE applications.

Recent advances in the field of functional polymeric biomaterials systems using a combination of synthetic and natural polymers enabled the fabrication of composite electrospun fibers characterized by promising and peculiar properties for tissue regeneration process.

### 2.3. Decellularized Extracellular Matrix (dECM)-Based Electrospun Fibers

In recent years, the research in scaffold engineering has shifted from using natural polymers to using the decellularized extracellular matrix (dECM) to obtain scaffolds mimicking native ECM [53]. Methods of decellularizing tissues have been extensively reviewed [54,55].

In general, decellularization procedures utilize mechanical, enzymatic, and chemical means to remove cellular material, that could induce an unwanted immune response and the rejection of dECM-based biomaterials and retain the intricate mixture of structural and functional elements of ECM.

However, the challenge of each decellularization method is to completely remove the cellular component and DNA content without removing and/or damaging structural components and functional ECM proteins such as glycosaminoglycans and growth factors [29].

Nowadays, experimental decellularization protocols vary widely between tissue and organ types, and optimization of the processes is often required in optimal decellularization of the native extracellular matrix. In addition, further processing of dECM allows the production of powders or hydrogels that can be further treated in order to obtain biomaterials suitable for regenerative applications.

Indeed, the use of dECM products provides an ideal microenvironment for cells, with ample biological and chemical cues necessary to regulate cell behavior [56]. In addition, dECM was able to support the correct phenotypic differentiation of progenitor cells and the maintenance of tissue-specific cell phenotypes.

The limitations of dECM products involve a low reproducibility derived from batch-to-batch variability across different donors, and limited capacity to tune properties, e.g., mechanical stability, porosity, stiffness, and degradability [26]. In order to improve and refine the physical-chemical properties of dECM systems, a viable solution is their combination with natural and/or synthetic polymers to create composite biomaterials.

The mechanical properties, as well as the internal architecture of the scaffold, may play an important role in the regeneration of complex tissue such as skeletal muscle tissue, influencing cell proliferation, alignment, and myogenic differentiation. For this reason, electrospinning may provide a promising method for the fabrication of dECM-based scaffolds allowing for the modulation of architecture and mechanical properties [3].

In particular, dECM from different tissues of origin may be processed in order to obtain a suitable product that can be directly loaded into an electrospinning polymeric solution to fabricate composite scaffolds that combine the versatility of polymeric materials and the biological complexity of natural ECM. Baguiera et al. [57] decellularized rat brains and blended them with gelatin before electrospinning. The obtained fibrous scaffolds provide a suitable microenvironment for mesenchymal stromal cell adhesion, proliferation, and survival. A similar approach was reported to obtain an electrospun scaffold composed of decellularized porcine cardiac tissue and poly(ethylene oxide) [58]. In another work, Gao et al. [59] reported the fabrication and characterization of an electrospun scaffold composed of a decellularized meniscus extracellular matrix and PCL.

Decellularized skeletal muscle has been used in a number of different forms, but few studies have used processed dECM using electrospinning techniques.

For example, Patel et al. [60] investigated the physical and mechanical properties of aligned nanofibers of decellularized muscle tissue and PCL. In vitro testing showed that the electrospun scaffold supports satellite cell growth, myogenic protein expression, and myokine production. This latter result suggests that dECM proteins provide the cues for attachment and growth of satellite cells onto the nanofibers, while the presence of PCL assures structural integrity and elasticity to the scaffold. A study developed a method to fabricate electrospun scaffolds from the decellularized skeletal muscle without the need for a carrier polymer is noteworthy [30]. The resulting scaffolds showed tunable physicochemical properties, including fiber alignment, while important extracellular matrix components for regeneration such as GAGs, were preserved.

## 3. Bioactivity and Biofunctionalization of Electrospun Scaffolds

In recent years, bioactive scaffolds obtained through the combination of polymeric materials and biomolecules attracted great attention due to the capability to express biological signals and, thus, to support cellular activity and promote tissue regeneration acting as molecules/drug delivery systems. The biomolecules range from growth factors, proteins, short peptides, genes, and enzymes, such that electrospun scaffolds can be functionalized largely and by several methods. Bioactive components can be both internalized (encapsulated) into the fibrous scaffold during the electrospinning and attached on the fiber surface in a postprocessing step. Some strategies are examined.

### 3.1. Bulk Biofunctionalization

The biomolecules can be introduced directly into fibers during the fabrication process. A simple method is to blend the bioactive molecules directly with the polymeric solution prior to the electrospinning process (blend electrospinning) [31]. In this way, bioactive components are dispersed into the electrospun scaffolds and can provide continued or controlled release of drugs/biomolecules for tissue regeneration. A drawback is the potential loss or alteration of the activity of the incorporated biomolecules caused by voltage or interaction with polymeric solution (e.g., in case of organic solvents such as chloroform, DMF, methanol, dichloromethane used for synthetic polymers such as PCL).

To overcome this inconvenience, loading biomolecules such as proteins or enzymes could require the use of more complex techniques, such as emulsion or coaxial electrospinning. These approaches enable to produce multiphase core-shell fibers, which allow a high loading capacity of bioactive molecules into the core and thus the preservation of their activity. In coaxial electrospinning, two different solutions are coaxially and simultaneous electrospun through different annular channels of a single needle. Furthermore, the peculiar coaxial structure is essential to control the release rate and maintain the mechanical and biological properties of the scaffold [61].

Emulsion electrospinning is a novel and simple technique to fabricate core-shell nanofibers, and either water-in-oil or oil-in-water emulsions can be electrospun to directly encapsulate hydrophilic or hydrophobic bioactive compounds into core-shell fibers, respectively [62].

Table 1 summarized some representative bioactive electrospun scaffolds characteristics in terms of polymeric matrices used, loaded biomolecules and the method of electrospinning used.

### 3.2. Surface Biofunctionalization and Click Chemistry

Surface biofunctionalization strategies aim to immobilize biomolecules such as proteins, peptides and polysaccharides, or bioactive drugs onto the surface of the electrospun fibers to modulate the interactions between biomaterial surfaces and biological systems. In order to promote the interaction between biomolecules and scaffold polymers, often it is necessary to perform surface activation. Relatively sophisticated surface modification can be accomplished via plasma treatment or wet chemical etching, which generate specific functional groups on the surface [11,32]. An ideal surface biofunctionalization process should be selective with easy reaction conditions avoiding the degradation of fiber, and assuring sufficient yields with a defined release profile of biomolecules without loss of their activity. This can be achieved via adsorption techniques, usually following the two fundamental strategies below.

- Physical adsorption is a simple approach that involves incubating the scaffold in a solution containing biomolecules. The biomolecules attach onto the scaffold surface owing to surface interactions, e.g., electrostatic forces, van der Waals forces, and hydrogen bonds.

In this context, electrospun nanofibrous PCL scaffolds combined with the osteogenetic growth factor bone, morphogenetic protein 2 (BMP-2), in association with chitosan by the layer by-layer (LbL) method have been shown to stimulate the regeneration of bone [71]. Layer-by-layer (LbL) is a simple and versatile technique for the realization of multimaterial coatings on the different substrate surfaces, which involves the sequential surface adsorption of alternating layers of oppositely charged polyelectrolytes [72]. Peptides and proteins chemically bound to polyelectrolytes, incorporated in polyelectrolyte multilayer architecture, have been shown to retain their biological activities [73].

Furthermore, the characteristics of electrospun systems such as fiber alignment, size, and porosity play a significant role in protein adsorption [74]. The main advantages are the simplicity of the procedure and the limited damage to fibers and biomolecules. In addition, the interaction could be relatively weak and the release of these biomolecules quick and uncontrolled.

- Chemical immobilization of biomolecules to the surface fibers is realized by the creation of a chemical bonding between functional groups of the components and those of bioactive molecules. Compared to physical adsorption, the covalent surface immobilization of biomolecules results in a more efficient coating; moreover, the bioactive components are retained over a longer period of time, promoting tissue regeneration [75]. In particular, an appropriate choice of polymers—biodegradable or nondegradable—allows the release rate of bioactive components to be controlled.

In this context, great attention is currently devoted to “click chemistry” since “click” reactions offer high reactivity, selectivity, and mild reaction conditions [34,76,77].

**Figure 2:**
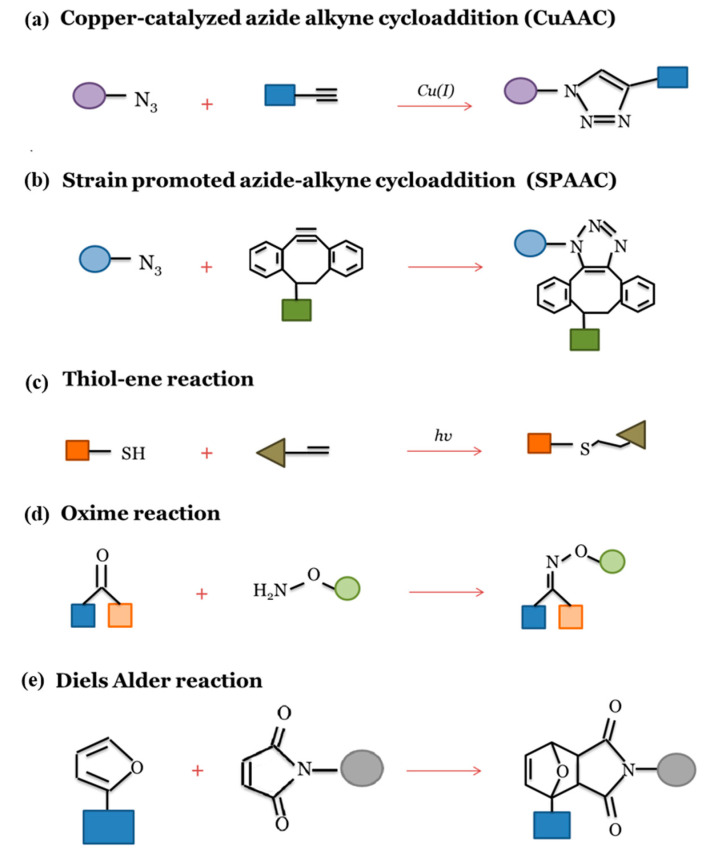
Schematic illustration of representative “click” reactions: (**a**) copper-catalyzed azide-alkyne cycloaddition (CuAAC); (**b**) strain promoted azide-alkyne cycloaddition (SPAAC); (**c**) thiol-ene reaction; (**d**) oxime reaction and (**e**) Diels Alder reaction.

The click chemistry approach was first introduced by Sharpless et al. [78], and the overview and classification of “click” reactions are reported in Figure 2.

In recent years, the use of ‘clickable’ building blocks, initiators, monomers, postmodifiers, and cross-linkers has led to the achievement of a wide variety of scaffolds promising in tissue engineering applications [34].

“Clickable” building blocks are common simple molecular subunits possessing one or more “clickable” groups for additional macromolecular construction and required to fabricate polymeric scaffolds with improved functions via “click” reactions. “Clickable” initiators are used to prepare polymers with chain-end ‘click’ functional groups. Instead, the direct polymerization of “clickable” monomers is needed for the development of polymers with multiple “clickable” sites enabling the further biomodification of scaffold. “Clickable” postmodifiers are recognized as effective tools for the direct functionalization of polymers with ‘clickable’ groups.

The modification of the electrospun fiber surface with biomolecules by click chemistry can be obtained through the direct electrospinning of functional polymers with reactive and clickable functional groups. For example, Lancuški et al. developed clickable nanofibrous scaffolds from a mixture of PCL-80K and PCL-2K with azide groups by electrospinning [79]. The surface of PCL-2K-azide presented clickable sites for CuAAC reaction (Figure 2a). Then, the obtained nanofibers could easily click onto desired biomolecules containing alkyne groups.

In another work, random and aligned (Dibenzyocyclooctynol) DIBO-terminated PLLA fibers were biofunctionalized with the Tyr-Ile-Gly-Ser-Arg (YIGSR) peptide after electrospinning using SPAAC click reaction (Figure 2b). The results showed that the aligned and biofunctionalized fibers enhanced neurite length and neural expression in mouse embryonic stem cells (mESC) compared to random fibers and to those without YIGSR functionalization [80].

The bioactive peptides and fluorescent molecules were bonded to the surface of electrospun PEU nanofibers by click reaction in aqueous media [81]. In particular, electrospun PEU nanofibers with “clickable” groups such as alkyne, azide, alkene, tyrosine-phenol, and ketone groups were successfully obtained. Depending on the “clickable” group, the functionalization of the PEU nanofibers via CuAAC, thiol-ene (Figure 2c), and oxime reactions (Figure 2d) can be performed [81].

In a recent work, polylactide (PLA)-based copolymers containing furan groups and triethylene glycol (TEG) were synthesized and electrospun to yield nanofibers [82]. After electrospinning, these nanofibers were conjugated with cyclic peptide, cRGDfK- maleimide via Diels-Alder reaction (Figure 2e). The presence of RGD peptide promoted cell adhesion and proliferation of L929 mouse fibroblasts suggesting that the proposed scaffolds were biocompatible and also provided a highly cytocompatible environment [82].

Furthermore, multiple click functional groups on electrospun fibers allow the attachment of different biomolecules. For example, Gly-Arg-Gly-Asp-Ser (GRGDS) and YIGSR peptides were introduced on the electrospun PCL surface via SPAAC and oxime reactions [83]. Modified GRGDS and YIGSR peptides, possessing an azide group and hydroxylamine, respectively, are introduced on the surface DIBO-PLC fibers in a one-pot reaction. The Schwann cell proliferation and attachment measurement suggest that biofunctionalized fibers are promising for peripheral nerve regeneration.

In another research, SPAAC, oxime, and CuAAC reactions were used to sequentially bond GRGDS, BMP-2 peptide, and dopamine on the electrospun DIBO-PCL scaffold surfaces [84]. This approach demonstrated a sequential and well-controlled bioactive component loading which could be useful to mimic the complex native ECM composition and activity in order to promote the regeneration tissue process.

Nowadays, thanks to the advances in click chemistry, clickable polymeric fibers may represent a major opportunity to manufacture suitable systems to bond various biomolecules with different approaches for tissue engineering applications.

## 4. Customized Functionalization by Click Chemistry of Composite dECM-Based Electrospun Scaffold for Skeletal Tissue Regeneration

Skeletal muscle is an important body-composition component in humans and plays a key role in voluntary movement and locomotion [85]. In addition, skeletal muscle is involved in other physiological processes, including thermogenesis, metabolism, and the secretion of numerous peptides for communication with other tissues [85]. For these reasons, the maintenance of skeletal muscle health is of vital importance. Although skeletal muscle is highly regenerative following injury or disease, endogenous self-regeneration is harshly impaired in the conditions of severe volume traumatic muscle loss (VML). Consequently, a growing demand arose in skeletal muscle TE to fully restore the structure and function of lost muscle [86].

The structure of muscle tissue is composed of oriented muscle fibers (myofibers), which are embedded into an extracellular matrix (ECM) consisting of many components such as collagen, glycoproteins, proteoglycans, and elastin [87].

Collagens form a network of intramuscular connective tissue (IMCT). The IMCT is typically organized in three layers: (i) the endomysium, representing the innermost layer that encloses individual muscle fibers; (ii) the perimysium bundling groups of muscle fibers; (iii) the epimysium enveloping the entire muscle. The IMCT contains various forms of collagens with types I and III being the most abundant [85]. The endomysium interfaces with the myofiber sarcolemma at a specialized basement membrane, which consists primarily of type IV collagen and laminin. Collagen type IV, a helical molecule, produces a network structure that with laminin, constitutes the basis of the basal lamina, directly linked to the sarcolemma of myofibers [85].

In addition, ECM, through its components such as GAGs, bind and store various growth factors which influence cell behavior and regulate cell proliferation, migration, and differentiation [87].

The skeletal muscle engineering approaches focus on the design and development of a fibrous scaffold with appropriate mechanical, morphological, and biofunctional properties to facilitate muscle growth and regeneration [87].

To this end, electrospinning is a forefront method to produce a fibrous scaffold that could mimic the structure and high anisotropic organization of native muscle tissue [88]. In particular, the composite electrospun scaffold could assure biocompatibility, biodegradability, controlled mechanical properties, and high porosity due to the synergistic effects of the different components.

Furthermore, advances in decellularization protocols of muscle tissue allow the preservation of the important components of ECM and the realization of potential dECM-based scaffolds [30,89,90,91] that could provide the correct support for myofiber development and the appropriate architecture to form muscle regeneration.

Some representative studies on electrospun biomaterials for skeletal muscle regeneration with the related principal outcomes are summarized in Table 2.

Despite the advantages of using dECM, the attachment of biomolecules onto its surface could be an interesting method of improving its biological potential. For example, the functionalization of dECM platforms with synthetic peptides with different cell adhesive sequences increased in vitro human umbilical vein endothelial cell adhesion compared to nonfunctionalized controls [96]. In addition, dECM was modified with a peptide (QK) mimicking vascular endothelial growth factor in order to enhance angiogenesis of endothelial cells. The functionalization was achieved using CuAAC click reaction, and the resulted triazole linkages seem not to compromise the integrity of dECM [97]. In another study, scaffolds composed of a skeletal muscle-derived decellularized extracellular matrix and myogenic factor insulin growth factor-1 (IGF-1) showed high in vitro cellular activities, including cell viability, proliferation, and differentiation. Moreover, in vivo implantation of dECM-based scaffold in the rabbit muscle defect model showed an acceleration of the novel muscle formation [98].

In addition, dECM scaffolds are known to exhibit poor biomechanical properties and control over their mechanical properties, and degradation is lacking [10,95]. A scaffold composed of dECM and synthetic polymers could be mechanically suitable to support functions of skeletal muscle.

Coupling electrospinning and click reactions to biofunctionalization surface electrospun scaffolds appears to be a valid method to design biofunctionalized fibrous scaffolds promising for skeletal muscle regeneration.

**Figure 3:**
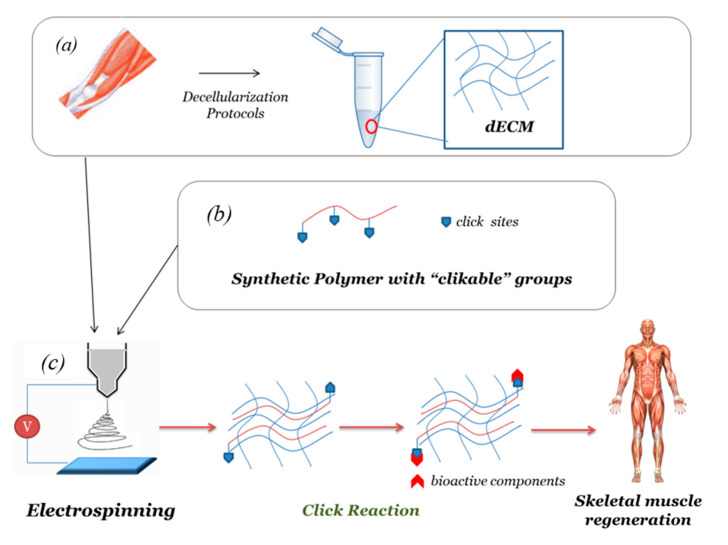
Manufacturing process proposal of composite scaffold for skeletal muscle regeneration: schematic representation. The main steps concern: (**a**) native skeletal muscle is processed to obtain a suitable dECM product; (**b**) synthetic polymers with “clickable” functional groups; (**c**) electrospinning of blend based on dECM (**a**) and the polymeric solution (**b**). The fibrous scaffold is biofunctionalized via click reaction in order to attach bioactive molecules promising for skeletal muscle regeneration.

In this context, a promising design strategy concerns the fabrication of composite fiber scaffolds composed of dECM of muscle tissue and synthetic polymer with targeted clickable functional groups. The main steps are reported in Figure 3.

In particular, scaffolds composed of fibers of decellularized ECM from skeletal muscle could mimic native muscle architecture and provide biological activity. Additionally, combining dECM with a synthetic polymer such as PCL provides mechanical stability to allow the tissue regeneration process [95]. In order to increase the bioactivity of the synthetic polymer component, this could contain clickable sites enabling further binding with appropriate biomolecules in muscle tissue regeneration.

After the electrospinning process, a click reaction such as cycloaddition between an azide and an alkyne is a suitable method to promote bioconjugation between synthetic polymer and biomolecules. It was demonstrated that azide- functionalized PCL [79] or alkyne- functionalized PCL [99] can be obtained.

Azide or alkyne groups are known for their stability to undergo click reactions. The presence of clickable sites on the surface scaffolds allows the binding with various bioactive biomolecules such as alkyne functionalized proteins or azide functionalized growth factors.

The chemical immobilization of azide conjugated epidermal growth factor (EGF) onto dibenzocyclooctyne (DBCO)-modified collagen surface through a strain-promoted azide-alkyne cycloaddition (SPAAC) reaction was reported by Lee et al. [100].

The protocol utilized consists of, first, the introduction of “clickable” functional groups to the collagen and EGF via an N-hydroxysuccinimide (NHS) ester reaction. The chemicals of the DBCO- and azide-containing NHS ester group were utilized for the modification of the collagen surface and EGF, respectively.

Afterwards, SPAAC reaction occurs between the azide group on the EGF and DBCO groups tethered on the collagen surface and generate stable triazole bonding, leading to the covalent immobilization of EGF onto the collagen surface.

This approach demonstrates the potential and useful application of copper-free click chemistry to combine growth factors to the ad hoc modified biomaterial surfaces. However, the selective modification of NHS ester reaction to the N-terminal primary amine group could improve the EGF bioactivity showed after the modification of all primary amine groups.

In an effort to better mimic physiologically relevant environments of skeletal muscle regeneration, growth factors such as FGF, PDGF, HGF and IGF, and short peptides can be used to biofunctionalize the scaffolds [87]. FGF, PDGF, and HGF promote the activation and proliferation of myogenic progenitor cells [101]. Insulin-like growth factor (IGF-I) plays an important role in all phases of satellite cell myogenesis by controlling the migration, proliferation, and differentiation of muscle satellite/progenitor cells [102].

In addition, the Arginine-Glycine-Aspartate (RGD) oligopeptide sequence that specifically recognizes and binds to integrin receptors is used to promote scaffold interaction with the cell surface. Many studies have tested the effectiveness of RGD peptide coated scaffolds for cell adhesion, and their influence on cell behavior with respect to tissue engineering could be incorporated into degradable matrices [103].

An attractive alternative concerns the realization of core-shell electrospun scaffolds. In particular, the creating of synthetic polymers-based systems incorporating dECM by the coaxial electrospinning technique could preserve the bioactivity of components dECM. In this approach, the biofunctionalization of the surface scaffold by click chemistry will assure cell adhesion and interaction, and the dECM-based core will assure an appropriate microenvironment for cell activity enabling skeletal muscle regeneration.

## 5. Conclusions

Electrospinning is a simple and versatile technique to produce polymeric fibrous scaffolds that are capable of mimicking the structure of the natural extracellular matrix (ECM) for a range of tissue engineering applications. In recent years, the decellularized extracellular matrix (dECM) has been demonstrated to be a suitable and promising starting material to fabricate composite electrospun scaffolds. In addition, the biofunctionalization of the electrospun fibers represents an interesting approach for the further exploration of the potential of electrospinning. In particular, the bioactivity of electrospun scaffolds could be assured by the covalent immobilization of biomolecules via click reaction in order to promote cell adhesion and proliferation. The combination of electrospinning and click reactions represents a promising strategy to design and develop leading and smart biomaterials systems for tissue engineering applications. In perspective, this strategy could be a valid starting point for obtaining customizable and intelligent biomaterial systems that are able to successfully guide the tissue regeneration process. In this context, we designed the fabrication of dECM-based composite electrospun scaffolds, and also, we proposed the surface biofunctionalization of these systems via click chemistry in order to modify the surface of the electrospun fibers with selected growth factors or peptides to guide skeletal muscle regeneration.

## Figures and Tables

**Table 1:** Bioactive electrospun scaffolds obtained with different methods of preparation, in chronological order.

Polymeric Component	Loaded Biomolecules	Method of Preparation	Reference
PLGA	bFGF	Coaxial electrospinning	[63]
PVA core PCL shell	GF loaded liposomes	Coaxial electrospinning	[64]
PCLC	VEGF	Emulsion electrospinning	[65]
PELCL core PELCL shell	VEGF PDGF	Coaxial electrospinning	[66]
PCL	bFGF	Emulsion electrospinning	[67]
PCL	VEGF	Blend electrospinning	[68]
PVA core PLA shell	CTGF	Coaxial electrospinning	[69]
PLA	PDGF	Coaxial electrospinning	[70]

Abbreviation: PLGA, poly(lactic-co-glycolic acid); bFGF, basic fibroblast growth factor; PVA, poly(vinyl alcohol); PCL, poly(ε-caprolactone); GF, growth factor; PLCL, poly(L-lactic acid-co-ε-caprolactone); VEGF, vascular endothelial growth factor; PELCL poly(ethyleneglycol)-b-poly(L-lactide-co-caprolactone); PDGF, platelet-derived growth factor; PLA, polylacticacid; CTGF, connective tissue growth factor.

**Table 2:** Some representative studies on electrospun biomaterials for skeletal muscle regeneration, in chronological order.

Electrospun Biomaterials	Experimental Model	Outcomes	Reference
PCL/collagen I	In vitro: Human skeletal muscle cells (hSkMCs)	Aligned PCL/collagen nanofibers significantly induced muscle cell alignment and myotube formation as compared to randomly oriented nanofibers	[47]
PLGA	In vitro: Murine myoblast cells (C2C12)	Aligned PLGA fibers control the myoblast elongation and alignment and encourage myoblast differentiation.	[92]
Chitosan/PCL	In vitro: Murine myoblast cells (C2C12)	Aligned chitosan-PCL nanofibrous scaffolds exhibited superior tensile strength compared to randomly oriented nanofibers and promoted muscle cell proliferation.	[93]
Chitosan/PVA	In vitro: Rabbit’s bone marrow (MSCs) In vivo: Adult New Zealand rabbit	Good cell viability, adhesion growth, and significant proliferation with less immune responses when the scaffold was implanted into the leg muscle of rabbit.	[51]
dECM from rabbit skeletal muscle	In vivo: Rabbit	The decellularization protocol of skeletal muscle tissue retains important ECM components. Electrospun scaffold derived completely from skeletal muscle dECM.	[30]
PLGA	In vitro: Murine myoblast cells (C2C12) In vivo: Mdx mice	Aligned PLGA fiber with larger diameter support enhanced alignment, growth, and differentiation of myoblasts. In vivo the optimized scaffolds seeded with primary myoblasts result in the formation of dystrophin-positive myofibers network.	[94]
PCL/dECM from bovine skeletal muscle	In vitro: Rat muscle precursor cells In vivo: C57/BL6 adult mice	Aligned nanofibers support satellite cell growth, myogenic protein expression, and myokine production. In vivo: myofiber regeneration was observed.	[60,95]

Abbreviation: PCL, poly(ε-caprolactone); PLGA, poly(lactic-co-glycolic acid); PVA, poly(vinyl alcohol).
